# ANN and Fuzzy Logic Based Model to Evaluate Huntington Disease Symptoms

**DOI:** 10.1155/2018/4581272

**Published:** 2018-03-11

**Authors:** Andrius Lauraitis, Rytis Maskeliūnas, Robertas Damaševičius

**Affiliations:** ^1^Department of Multimedia Engineering, Kaunas University of Technology, Studentų 50, Kaunas, Lithuania; ^2^Center of Real Time Computing Systems, Kaunas University of Technology, K. Baršausko 59, Kaunas, Lithuania; ^3^Department of Software Engineering, Kaunas University of Technology, Studentų 50, Kaunas, Lithuania

## Abstract

We introduce an approach to predict deterioration of reaction state for people having neurological movement disorders such as hand tremors and nonvoluntary movements. These involuntary motor features are closely related to the symptoms occurring in patients suffering from Huntington's disease (HD). We propose a hybrid (neurofuzzy) model that combines an artificial neural network (ANN) to predict the functional capacity level (FCL) of a person and a fuzzy logic system (FLS) to determine a stage of reaction. We analyzed our own dataset of 3032 records collected from 20 test subjects (both healthy and HD patients) using smart phones or tablets by asking a patient to locate circular objects on the device's screen. We describe the preparation and labelling of data for the neural network, selection of training algorithms, modelling of the fuzzy logic controller, and construction and implementation of the hybrid model. The feed-forward backpropagation (FFBP) neural network achieved the regression *R* value of 0.98 and mean squared error (MSE) values of 0.08, while the FLS provides a final evaluation of subject's reaction condition in terms of FCL.

## 1. Introduction

Huntington disease (HD) is a progressive genetic neurodegenerative disorder causing involuntary movement and cognitive problems that significantly affect daily life of HD patients. HD affects about 1 in 10,000 to 20,000 people of European (Caucasian) descent [1], though in some isolated populations it is much higher. HD reduces life expectancy due to heart disease, pneumonia, physical injury from falls, and suicide. The most visible symptom of HD is chorea, which consists of jerky, involuntary movements of the upper and lower extremities, face or body, and occurs in about 90% of patients at some stage of their illness [2]. Other symptoms include behavioural problems, cognitive impairment, psychiatric disorders, and dementia, which have a serious impact on daily living of a patient and often result in hospitalization. The societal and financial cost of HD on health and social care systems is significant and is estimated to be £195 million per year in the UK alone [3].

HD is currently incurable so most of the current research in this area focuses on identifying the deficits at the early stage of the disease, to benefit from future medical interventions that may help delaying the progress of the disease [4, 5]. This is also the case of the work presented in this paper. Traditional HD research often include magnetic resonance neuroimaging (MRI) measures of striatum and white matter volume, CAG repeat length in chromosome analysis, age, and striatal atrophy [6, 7]. Moreover, medical personnel and doctors who have experience in caring after HD patients and knowing that disease is cureless are not usually motivated to conduct scientific research themselves or to support multidiscipline (e.g., bioinformatics) investigations.

Any scientific result (device, technology, and theoretical model) that could contribute towards improvement of daily life of HD patient's and help to monitor or predict the progress of the disease can be useful for both doctors and HD patients.

The problematics of data prediction evolved with the rise of artificial intelligence (AI) and machine learning (ML) methods and algorithms. Artificial neural networks (ANN) such as multilayer perceptron (MLP) can be used for classification of accelerometer-based tremor signals invoked by Parkinson patient's involuntary movements [8]. Prediction of Parkinson disease onset by adapting radial basis function neural network (RBFNN) for tremor activity data recorded via stimulation electrodes using electromyography (EMG) signals is described in [9]. Dynamic neural network (DNN) is used to detect time-varying occurrences of tremor and dyskinesia from time series data acquired from EMG sensors and triaxial accelerometers worn by Parkinson patients [10]. Another approach of designing a prediction model for Parkinson's disease uses a decision tree and Iterative Dichotomiser (ID3) methods to analyze data collected from HD symptoms such as trembling in the legs, arms, hands, impaired speech articulation, and production difficulties [11]. Hybrid models combine different AI and ML approaches for reproducing intelligent human reasoning process [12]. By using information fusion, hybrid models combine heterogeneous ML approaches and improve quality of reasoning for complex regression and classification problems [13]. Neurofuzzy systems combine neural network and fuzzy logic paradigms to avoid the limitations of neural network explanations to reach decision and limitations of fuzzy logic to automatically acquire the rules used for making those decisions [14]. Fuzzy expert systems such as neurofuzzy system (ANFIS) can be applied in assessment of Parkinson's disease with a noninvasive screening system for quantitative evaluation and analysis by using amplitude, frequency, spectral characteristics, and trembling localization parameters of input data [15]. Hybrid model is adapted in designing a decision support system (DSS) for the intelligent identification of Alzheimer where neurofuzzy system explores approximation techniques from neural networks to find the parameter of a fuzzy system [16]. Hybrid systems are also used as a classifier fusion strategy (Bayesian, SVM, k-nearest neighbours) in the prevalence of age-related diseases like Alzheimer's and dementia [17], diagnostics and measurement [18] with wavelet transform (WT) and norm entropy feature extraction methods. The DSS that uses MLP and RBFNN is applied for monitoring patients with neurological disorders [19]. The data is collected using noninvasive smart devices (modified mouse and 3-axis accelerometer sensor). Integration of neurofuzzy networks and information fusion for multimodal human cognitive state recognition is described in [20]. Projection-based learning for metacognitive radial basis function network (PBL-McRBFN) is applied to predict Parkinson's disease [21]. Other hybrid systems and applications include nonlinear adaptive system, which fuses brain and gait information algorithmically using multistate Markov model [22]. Accurate Parkinson disease diagnosis model based on cluster analysis uses random tree, classification and regression tree (C-*RT*), ID3, binary logistic regression, k-NN, partial least square regression (PLS), support vector machines (SVM) [23], and fuzzy c-means clustering (FCM) [24].  Table 1 provides a summary of methods used by other authors.

Our previous work included the development of text input-based system for evaluating the condition of Huntington's patients [25]. The use of ANN for predicting the functional capacity of a Huntington's patient was proposed in [26].

The aim of this paper is to create a computerized behavioural model, which predicts an impaired reaction condition for HD patients. We develop a mobile application to collect a dataset using finger touch coordinates and reaction time features extracted from test subjects (healthy and HD patients); create an ANN to predict the functional capacity level and fuzzy logic system (FLS) to determine the reaction condition (stage) for individual person; combine ANN with FLS into a hybrid model to predict the impaired reaction condition for HD patients; and simulate an experimental setup for test subjects to perform a provided exercise (test) at the different moments in time in order to predict a possibly impairing reaction condition with the help of the proposed hybrid model.

## 2. Materials and Methods

### 2.1. Subjects

The study included ten (10) Huntington disease (HD) patients living in Lithuania. Each HD patient agreed to participate and allowed the data collected during the test to be used for scientific purposes. Every HD patient fall in the early clinical descriptor category of Huntington disease, that is, I and II stages according to Shoulson–Fahn evaluation system [27]. Such HD patients have hand tremors, body movement distractions, but are capable to perform the test on a mobile application without extra help, for example, from medical personnel, nurses, or family members. Other ten (10) participants were healthy people with no signs of any neurological or neurodegenerative disorder.

### 2.2. Procedure

The test can be performed using various mobile devices that support Android OS. The mobile application randomly generates circular shape objects (2, 3, and 5 circles at time) of particular color that are generated on the mobile device's screen. Each circle is located in different positions of the screen, thus no possible collisions (overlapping) between two particular circles are possible. An active circle that needs to be touched is marked by a black contour so as to differ from other objects.

The subjects are instructed to touch every object, starting from first in sequence, by finger as close to center and as quickly as possible. When subject finishes the test, collected data is stored in external mobile device storage and sent to the database using the internet connection.

### 2.3. Dataset

The collected dataset consists of 3032 data examples collected from 20 test subjects (10—healthy and 10—HD patients). The dataset (see a sample in Table 2) contains the ground truth coordinates of the generated object, the coordinates of subject's touch, subject's reaction time, subject's label, and the marker of Huntington's disease.

### 2.4. Feature Extraction and Class Labelling

The subject's reaction time (*rt*) and the Euclidian distance between the two points of true and touched positions (*delta*) serve as features which are incorporated as input variables to ANN. We assume that smaller *rt* and *delta* values indicate better functional capacity level. The bigger *delta* value can show stronger hand tremoring, whereas higher *rt* value is an indicator of body stagnancy.

**Figure 1:**
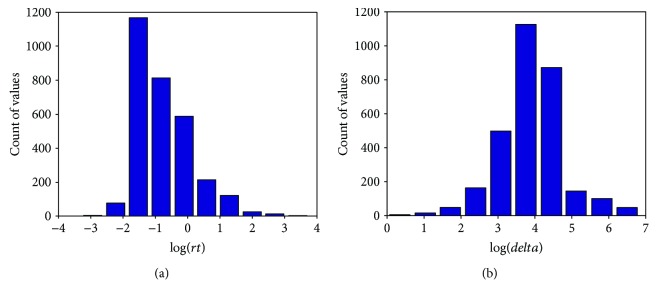
Statistical distribution of log-transformed *rt* (a) and *delta* (b) values.

The statistical analysis of the *rt* and *delta* values has revealed that the values are not normally distributed, but after the applying the log transformation, which is commonly used in regression analysis of biological data with highly skewed distribution [28], the values become normal as confirmed by visual inspection in Figure 1 and skewness *γ* and kurtosis *κ* tests (*γ*_*rt*_ = 1.046, *κ*_*rt*_ = 4.239 and *γ*_*delta*_ = 0.028, *κ*_*delta*_ = 4.779). For data samples greater than 300, values |*γ*| < 2 and |*κ*| < 7 are considered as acceptable for normality [29].

**Figure 2:**
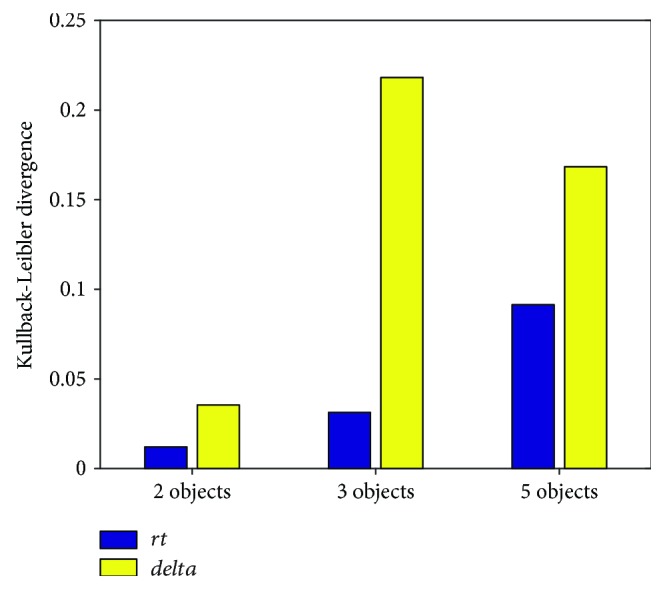
Feature ranking according to the Kullback–Leibler distance.

To analyze the power of *rt* and *delta* values to correctly predict the healthy or sick state of the subject, we have performed feature evaluation using the relative entropy (also known as the Kullback–Leibler distance or divergence) criterion, considering different number of objects presented at the screen. The results are presented in Figure 2. In all cases, *delta* feature has larger discriminative power than *rt*, and the features from 3 and 5 objects test are more statistically discriminative.

### 2.5. ANN for Functional Capacity Level Prediction

We have analyzed the following neural network models: (1) feed-forward backpropagation (FFBP); (2) feed-forward time delay neural network (FFTD); (3) cascade-forward backpropagation (CFBP); (4) nonlinear autoregressive exogenous model (NARX); (5) Elman neural network; (6) layer recurrent neural network (RNN); and (7) generalized regression neural network (GRNN).

FFBP is a simple neural network without any cycle connections between neurons [30]. FFTD has no internal state and adds delayed copies as other inputs as an input signal to obtain time-shift invariance [31]. In CFBP, the input values calculated after every hidden layer are backpropagated and the weights adjusted [32]. NARX have a limited feedback, which comes only from the output neuron rather than from hidden layer [33]. Elman network additionally has context units, which are connected to the hidden units, thus providing the network with memory [34]. RNN represent an architecture where connections between units form a directed cycle [35]. GRNN has only one (smoothness) parameter, and its convergence is guaranteed; fast and stable [36].

Each neural network has 2 inputs (*rt*, *delta*) and 1 output (Y). Neural network is composed of single neurons that are treated as a simple unit carrying signals (data) to each other or different layers via transfer functions, which correspond to sum of input signal. Training function is the optimization algorithm used for finding global minimum of a function. The outputs of ANN are class labels for determining the functional capacity of a person (the larger value indicates that a person is more capable to do motoric activities). Such scenario imitates the TFC scale measurement system for Huntington disease patients presented in Table 3 [27].

Table 4 illustrates the setup for analyzed ANN models with their parameters.

### 2.6. Training and Testing

The dataset was randomly divided into 3 sets: training, validation, and testing. Training set uses all samples from 70% of users. Validation set (15%) is used to measure network generalization and to stop training when necessary. Testing set (15%) provides independent performance of the network afterwards. We also analyzed a different partition of the dataset (40% for training, 30% for validation, and 30% for testing); however, there were no significant differences in the performance of ANN.

Overfitting was prevented by using the early stopping technique, which controls error on the validation set which is monitored during training process: when error increases for a specified number of iterations then the training is stopped and the weights and biases at the minimum of the validation error are returned.

For each neural network model, we have repeated the training and testing process for 20 times in order to allow calculation of statistical characteristics (mean, standard deviation) of ANN performance measures and to perform statistical comparison.

### 2.7. Reaction Stage Determination Using Fuzzy Logic System (FLS)

The aim of the FLS system is to determine the reaction stage of a patient (test subject) according to some predefined parameters. The FLS consists of three main parts: fuzzification block, inference mechanism, and defuzzification block. Membership functions, linguistic variables are created in fuzzification module. Inference engine is responsible for applying logical rules (fuzzy rule base) to the knowledge base and deduce new knowledge. Defuzzification module converts all the fuzzy terms created by the rule base of the controller to crisp terms (numerical values). The FLS uses triangular membership Mamdani-type functions with fuzzy set inference mechanism (minimum implication, maximum aggregation, minimum AND operator, maximum OR operator) and centroid defuzzification method.

The parameters of the FLS are derived from the ANN output corresponding to the functional capacity level, so in the FLS design process, the model input and output values need to be considered accordingly. There are three input and one output variable in the FLS. The input parameters are *AVG1*, *AVG2*, and *AVG3*, which correspond to the average of ANN output values when test subject is working with two, three, and five objects, respectively. All three inputs can have values in range [0; 10].

The linguistic variables (terms) for *AVG1*, *AVG2*, and *AVG3* are

1. *LOW* [0 2 4];
2. *AVERAGE* [3.6 5.5 7];
3. *HIGH* [6.6; 8.5 10].

The model has one output parameter *ReactionStage* can have one of five values: close to peaks 1, 3, 5, 7, or 9, that is, each peak corresponds to particular linguistic variable of *ReactionStage*. The terms for output parameter *ReactionStage* are

1. *ADVANCED* [0 1 2];
2. *LATE* [1.5 3 4];
3. *AVERAGE* [3.5 5 6];
4. *EARLY* [5.5 7 8];
5. *HEALTHY/PRECLINICAL* [7.5 9 10].

The FLS rule base is formed from 27 fuzzy rules.  Table 5 illustrates the principles of constructing fuzzy rule base. These can be interpreted as general fuzzy IF-THEN rules containing only fuzzy logical AND operators, for example,

IF *AVG1* is LOW AND *AVG2* is LOW AND *AVG3* is LOW

THEN *ReactionStage* is ADVANCED.

## 3. Proposed Hybrid Model

**Figure 3:**
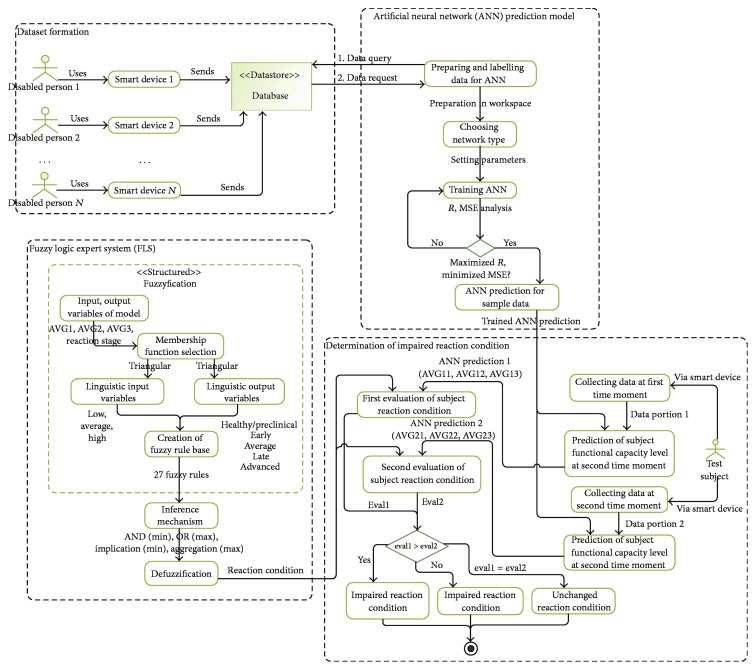
Schema of prototype hybrid model to forecast impaired reaction condition.

The hybrid model (see Figure 3) is composed of four sub models: (1) dataset formation; (2) ANN prediction model; (3) fuzzy logic expert system (FLS); and (4) decision module for determination of person's condition.

During dataset formation, test subjects (under the supervision of a healthy person—a medical doctor or a nurse) use smart devices to perform reaction and accuracy test experiments with their fingers. The collected data is stored in the database. The ANN submodel predicts the functional capacity level of a person using the data from the database. The network is trained by observing regression (*R*), that is, correlation measurement between outputs and targets and mean squared error (MSE) values. Once the network is trained, it can make predictions on new sample data. Finally, to evaluate the reaction condition of a test subject, the test session is repeated at a different time and the ANN predictions are aggregated, and the reaction stage of a person is evaluated using a fuzzy rules system.

## 4. Experimental Results

The hybrid model was implemented with MATLAB Neural Network and Fuzzy Logic Toolbox software (MathWorks Inc.). The results of regression and comparison of the prediction results of the analyzed ANN models is presented in Table 6, whereas the performance of neural networks in terms of means and 95% confidence intervals of *R* and MSE is given in Table 7. The “TFC” field indicates the ground truth evaluation of the patient state provided by a medical neurologist expert according to the TFC scale. The *R* metric measures the correlation between output and targets, whereas the MSE metric is the average squared difference between outputs and targets.

**Figure 4:**
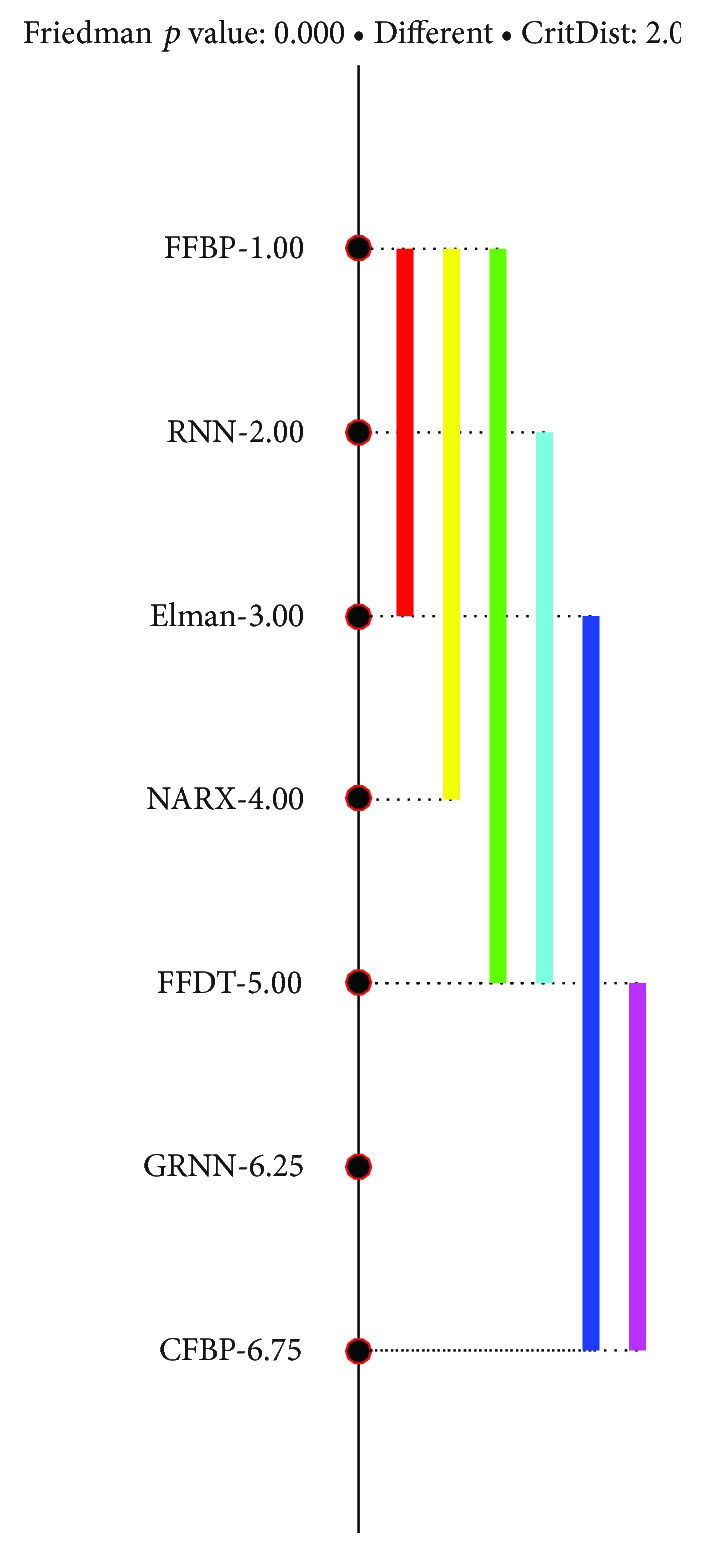
Results of Nemenyi test on performance (MSE) of ANN models.

Nonparametric Friedman test was conducted to compare the performance results (MSE) among ANN models. Results show that there is a significant difference in performance among all ANN models (chi-square = 133.15; *p* = 2 · 10^−26^). Posthoc Nemenyi tests further reveal that the performance of FFBP is the best among all ANN models (Figure 4).

**Figure 5:**
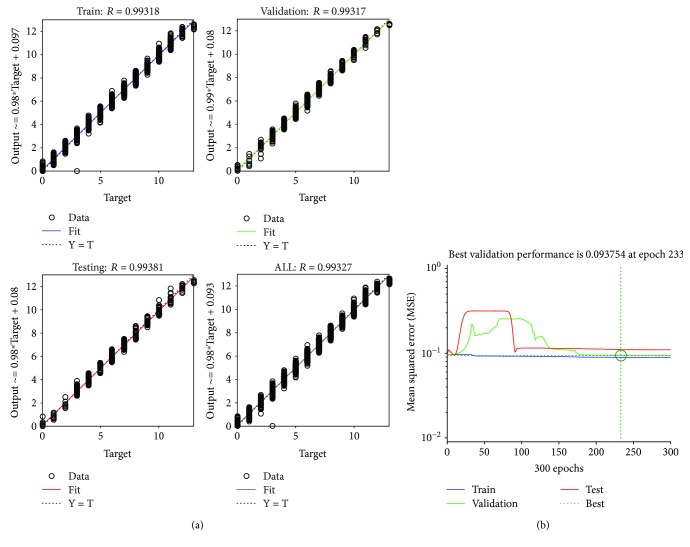
FFBP performance evaluation using *R* (a) and MSE (b) metrics.

Figure 5 shows an example of FFBP best performance equal to *R* = 0.993 and MSE = 0.094 on the validation set.

Table 8 illustrates impaired reaction condition simulation example on a single test subject using the FLS system. In order to make comparison, data samples were collected at different time moments. Feature (*rt1*, *delta1*, *rt2*, and *delta2*) values are presented in all three modes (10 attempts), thus giving two separate ANN (in the example provided, FFBP model was used) prediction outputs, which are used to calculate average values and evaluate the reaction condition in the FLS.

## 5. Conclusions

We have presented an actual experimental framework to assess finger-tapping tests performed by patients suffering from the Huntington's disease (HD). The proposed model was validated using a dataset of 3032 data records collected from 20 test subjects (both healthy and HD patients). The reaction condition was determined using the developed Mamdani Type-1 fuzzy logic expert system (FLS) with 3 input (3 linguistic variables), 1 output (5 linguistic variables), triangular membership functions, and 27 fuzzy rules base.

We describe an architecture that combines several artificial neural networks (ANN) of different type (FFBP, FFDT, CFBP, NARX, Elman, RNN, and GRNN) to create a hybrid (neurofuzzy) model, which integrates feature extraction, prediction, and classification routines to forecast the impaired reaction condition for HD patients. The best results were achieved using the feed-forward backpropagation (FFBP) neural network model, which predicts the total functionality capability (TFC score) with high performance results, that is, it has obtained regression *R* value not less than 0.98 and mean squared error (MSE) values of 0.08, while FLS evaluates several measurements taken time apart to provide a final evaluation of the subject's reaction condition.

Future work will focus on the validation of the proposed system using a larger dataset, which includes the data collected from the Parkinson's and Alzheimer's patients as well, the analysis and use of more sophisticated finger-tapping features, and the comparison of the ANN results with those of SVM regression.

## Figures and Tables

**Table 1:** Comparison of various ML methods adapted for neurodegenerative disorders such as Huntington or Parkinson disease to solve prediction and classification problems.

Work ref.	ML method	Learning approach	ML problem	Size of data set	Number of test subjects	Target group	Involve HD patients
[8]	ANN, MLP	Supervised	Classification	—	21	PD, healthy	No
[9]	RBFNN	Supervised	Regression	—	—	PD	No
[10]	DNN	Supervised	Classification	—	12	PD (8), healthy (4)	No
[11]	Decision tree, ID3	Supervised	Classification	195	31	PD (23), healthy (8)	No
[15]	Adaptive neurofuzzy	Hybrid		100	—	PD	No
[16]	Neurofuzzy system	Hybrid		—	—	ALS	No
[17]	Fusion of classifiers (Bayesian, SVM, k-nearest neighbor)	Hybrid		640	ALS (13), PD (15), HD (16), healthy (16)	ALS, PD, HD	Yes
[18]	Neurofuzzy system	Hybrid		—	—	Only survey was done	No
[19]	ANN + MLP, RBFNN	Hybrid		—	—	PD	No
[20]	Neurofuzzy system	Hybrid		—	—	—	No
[21]	PBL-McRBFN	Supervised	Classification	22,283	72 (50 PD, 22 healthy)	PD, healthy	No
[22]	Multistate Markov model	Hybrid		2500	72 (82 PD, 62 healthy)	PD, healthy	No
[23]	Random tree, (C-*RT*), ID3, binary logistic regression, k-NN, (PLS), (SVM)	Supervised	Classification	195	31 (23 PD, 8 healthy)	PD, healthy	No
[24]	FCM	Unsupervised	Clustering	195	—	PD	No

**Table 2:** Collected data from mobile application (random sample data of 5 records).

*x*	*y*	*xt*	*yt*	nC	*rt*	*delta*	User	IsSick
126	871	125	872	5	1.665	1.414	1	0
411	403	390	408	3	3.886	21.587	1	0
243	609	299	592	3	0.573	58.523	2	1
580	377	618	449	5	0.545	81.413	2	1
501	634	437	585	2	0.741	56.436	3	1

*x,y*: screen coordinates of the center of circular object to touch; *xt*, *yt*: screen coordinates of user touch; nC: number of circular objects rendered on the device screen; *rt*: user's reaction time in seconds; *delta*: the Euclidean distance between object's center and touch position. User: user ID; IsSick: indicates if test subject has Huntington disease (1 yes, 0 otherwise).

**Table 3:** Total functional capacity score (TFC) and its relationship to Shoulson–Fahn stages and clinical descriptors [27].

Descriptor	TFC	Stage
Early	11–13	I
	7–10	II
Moderate or mid	4–6	III
Advanced or late	1–3	IV
	0	V

**Table 4:** Summary of different neural network models and their configuration parameters.

Network	Hidden layer (neurons)	Transfer function	Training function	Number of weight elements	Time delay
FFBP	1 (10)	Log-sigmoid, linear	Gradient descent with adaptive learning rate backpropagation	41	−
FFTD	1 (10)	Tan-sigmoid	Levenberg–Marquardt	101	+
CFBP	1 (10)	Tan-sigmoid	Levenberg–Marquardt	43	−
NARX	1 (10)	Tan-sigmoid	Levenberg–Marquardt	81	+
Elman, RNN	1 (10)	Tan-sigmoid	Levenberg–Marquardt	141	+
GRNN	1 (size of dataset)	Radial basis, linear	Levenberg–Marquardt	800	−

**Table 5:** FLS rule base (5 random examples chosen for each reaction stage).

AVG1	AVG2	AVG3	Reaction stage
HIGH	HIGH	HIGH	HEALTHY/PRECLINICAL
HIGH	LOW	HIGH	EARLY
AVERAGE	AVERAGE	AVERAGE	AVERAGE
LOW	LOW	HIGH	LATE
LOW	LOW	LOW	ADVANCED

**Table 6:** Regression and prediction result comparison of different ANN models.

Functional capacity level predictions (data sample of 10 records from 1 test subject)										
*rt*	2.65	0.25	0.67	0.26	0.24	0.78	0.4	0.29	0.34	0.25
*delta*	22.36	108.3	87.8	267	60.1	20.8	37	113	41.4	68.8
TFC	8	3	4	1	5	8	7	3	7	5
FFBP	8.02	3.31	3.90	1.32	5.39	8.55	7.25	3.19	6.85	4.83
FFTD	7.99	3.26	3.94	0.97	5.41	8.51	7.24	3.15	6.83	4.87
CFBP	7.94	3.32	3.59	1.28	5.45	8.84	7.15	3.16	6.83	4.87
NARX	8.06	3.28	3.83	1.20	5.43	8.81	7.24	3.16	6.85	4.87
Elman	8.01	3.27	3.87	1.19	5.40	8.79	7.30	3.16	6.88	4.84
RNN	7.97	3.28	3.86	1.15	5.42	8.54	7.27	3.15	6.87	4.87
GRNN	8.20	2.92	3.86	0.99	5.18	8.93	6.83	2.91	6.88	4.87

**Table 7:** Performance comparison of analyzed ANN models.

Neural network model	Mean *R*	95% confidence intervals of *R*	Mean MSE	95% confidence intervals of MSE
FFBP	**0.9876**	**[0.9871, 0.9880]**	**0.0809**	**[0.0782, 0.0835]**
FFTD	0.9861	[0.9855, 0.9867]	0.0906	[0.0865, 0.0948]
CFBP	0.9827	[0.9787, 0.9868]	0.1125	[0.0860, 0.1389]
NARX	0.9868	[0.9868, 0.9869]	0.0858	[0.0855, 0.0860]
Elman	0.9868	[0.9868, 0.9868]	0.0857	[0.0856, 0.0857]
RNN	0.9870	[0.9870, 0.9870]	0.0845	[0.0845, 0.0845]
GRNN	0.9849	[0.9841, 0.9858]	0.0977	[0.0926, 0.1029]

**(a):** 

Feature	Mode 1 2 objects		Mode 2 3 objects			Mode 3 5 objects				
Dataset formation: data portion 1										
*rt1*	4.75	8.05	5.30	2.27	7.09	1.48	6.58	6.33	2.29	1.82
*delta1*	3.32	2.99	4.05	19.09	0.31	19.15	0.51	19.42	5.95	10.50
Artificial neural network (ANN) prediction model: ANN prediction 1										
	8.94	8.00	8.43	6.68	8.99	6.79	9.00	5.46	9.03	8.10
Dataset formation: data portion 2										
*rt2*	8.62	8.96	1.89	6.60	9.41	1.48	6.58	6.33	2.29	1.82
*delta2*	1.42	9.78	16.99	19.94	0.89	10.85	17.27	18.18	16.90	17.57
Artificial neural network (ANN) prediction model: ANN prediction 2										
	8.01	7.94	7.00	5.10	8.01	7.26	7.01	6.93	5.99	7.04

**(b):** 

Fuzzy logic expert system (FLS)									
AVG11	AVG12	AVG13	AVG21	AVG22	AVG23	eval1	eval2	Condition 1	Condition 2
*8.47*	8.03	7.67	7.98	6.70	6.84	9.00	7.00	Healthy/Preclinical	Early
*Conclusion:* Impaired reaction condition.									

## Additional Points

*Human Studies.* Research on human subjects was approved by the Institutional Review Board of the Faculty of Informatics of Kaunas University of Technology.
